# Recombinant Production of Bovine α_S1_-Casein in Genome-Reduced *Bacillus subtilis* Strain IIG-Bs-20-5-1

**DOI:** 10.3390/microorganisms13010060

**Published:** 2025-01-02

**Authors:** Lennart Biermann, Lea Rahel Tadele, Elvio Henrique Benatto Perino, Reed Nicholson, Lars Lilge, Rudolf Hausmann

**Affiliations:** 1 Institute of Food Science and Biotechnology, Department of Bioprocess Engineering, University of Hohenheim, Fruwirthstraße 12, 70599 Stuttgart, Germany; lennart.biermann@uni-hohenheim.de (L.B.); lea.tadele@uni-hohenheim.de (L.R.T.); eperino@uni-hohenheim.de (E.H.B.P.); rudolf.hausmann@uni-hohenheim.de (R.H.); 2 Motif FoodWorks, Inc., 27 Drydock Ave, Boston, MA 02210, USA; reed.a.nicholson@gmail.com

**Keywords:** *Bacillus subtilis*, genome reduction, alternative protein sources, α_S1_-casein, microbial expression systems, food proteins, sustainable food production

## Abstract

Background: Cow’s milk represents an important protein source. Here, especially casein proteins are important components, which might be a promising source of alternative protein production by microbial expression systems. Nevertheless, caseins are difficult-to-produce proteins, making heterologous production challenging. However, the potential of genome-reduced *Bacillus subtilis* was applied for the recombinant production of bovine α_S1_-casein protein. Methods: A plasmid-based gene expression system was established in *B. subtilis* allowing the production of his-tagged codon-optimized bovine α_S1_-casein. Upscaling in a fed-batch bioreactor system for high cell-density fermentation processes allowed for efficient recombinant α_S1_-casein production. After increasing the molecular abundance of the recombinant α_S1_-casein protein using immobilized metal affinity chromatography, zeta potential and particle size distribution were determined in comparison to native bovine α_S1_-casein. Results: Non-sporulating *B. subtilis* strain BMV9 and genome-reduced *B. subtilis* strain IIG-Bs-20-5-1 were applied for recombinant α_S1_-casein production. Casein was detectable only in the insoluble protein fraction of the genome-reduced *B. subtilis* strain. Subsequent high cell-density fed-batch bioreactor cultivations using strain IIG-Bs-20-5-1 resulted in a volumetric casein titer of 56.9 mg/L and a yield of 1.6 mg_casein_/g_CDW_ after reducing the *B. subtilis* protein content. Comparative analyses of zeta potential and particle size between pre-cleaned recombinant and native α_S1_-casein showed pH-mediated differences in aggregation behavior. Conclusions: The study demonstrates the potential of *B. subtilis* for the recombinant production of bovine α_S1_-casein and underlines the potential of genome reduction for the bioproduction of difficult-to-produce proteins.

## 1. Introduction

The demand for alternative animal-free foodstuffs is currently on the rise. Consequently, new strategies need to be found for sustainable agriculture and the associated demand for alternative protein sources [1]. In this context, bioproduction technologies offer a promising solution approach, particularly in milk protein production, as a scalable and cost-effective method for alternative protein production [2,3].

Bovine milk provides one of the most important sources of proteins, essential fatty acids, carbohydrates, vitamins, and minerals [4,5]. In terms of protein availability, casein represents around 80% of the total protein amount in bovine milk. Caseins are secretory proteins, which are post-translationally modified by phosphorylation (α_S1_-, α_S2_-, and β-casein) of serine residues and glycosylation (κ-casein) of threonine residues [6,7]. These proteins are characterized by their ability to form colloidal nanostructures called micelles—a key feature for many of their applications [8,9,10]. Its natural origin and non-toxicity make it also an ideal candidate for encapsulating and protecting pharmaceuticals, ensuring controlled release and enhanced bioavailability of drugs [11,12].

However, the post-translational modifications (PTMs) and high aggregation propensity make microbial casein bioproduction difficult. Microbial hosts often lack the necessary machinery to perform PTMs such as phosphorylation, which impairs the solubility and functionality of recombinant proteins [13]. In microbial expression systems, this often leads to the formation of inclusion bodies, in which the proteins are misfolded and biologically inactive, making their recovery and purification difficult [13,14]. The formation of micelles, which is crucial for the function of caseins in dairy products, is another challenging issue [15].

Nevertheless, the expression of α-, β-, and κ-casein was previously carried out in microbes such as *E. coli*, *S. cerevisiae*, and *P. pastoris* [16,17,18,19]. In most studies, only the expression of these proteins was detected, while titers and yields were rarely reported. Exemplarily, Choi and Jiménez-Flores [19] reported a titer of 245 mg/L of bovine β-casein using *P. pastoris* strain GS115 in a five-day culture in buffered minimal methanol medium for the production. Wang et al. [20] achieved in a cultivation with artificial wheat straw hydrolysate medium, using the *E. coli* strain BL21(DE3) as the production strain, bovine α_S1_-casein titers of 1.13 g/L. However, although those microbes are used by various start-ups, among others, for the production of animal-free, but animal-like foods from purified proteins [21], challenges in production inefficiency due to insufficient expression systems and adaptation of posttranslational target protein modifications, identification of cost-effective downstream processing strategy including purification methods such as gel filtration, ion exchange chromatography, hydrophobic chromatography and affinity chromatography, and food safety evaluations in terms of allergenicity or toxicity to consumers by carrying out a risk assessment (genetic engineering, stability of the product, dietary nutrition, toxicity, sensitization) are still present [3,21].

In this context, the application of *B. subtilis*, as a super-secreting cell factory [22,23], allows the bioproduction of different recombinant proteins. *B. subtilis* also appears to be a promising host for the recombinant expression of food additives, as some of the products are generally recognized as safe (GRAS), which may also improve public acceptance of microbially produced food proteins as a key to their success [24]. In this context, the formation of target proteins and localization/accumulation in inclusion bodies using a microorganism that is harmless to humans might be another beneficial strategy for the microbial production of food additives [25,26]. In addition, *B. subtilis* allows the formation of high biomass in adapted high cell density fed-batch bioreactor fermentation processes using sporulation-deficient production strains [27,28]. For instance, Tran et al. [29] showed the potential of *B. subtilis* as a production strain for recombinant proteins using the strong IPTG-inducible P_grac100_ promoter system, leading to 20.9% of the target protein compared to the total intracellular protein amount after 12 h of induction time [29]. In contrast, Scheidler et al. [26] developed an efficient amber suppression system in *B. subtilis* using IPTG as an inducer, enabling the production of proteins with non-canonical amino acids. In this way, a notable yield of 2 mg/L could be achieved for the light chain variable domain of a murine monoclonal antibody fragment (MAK33-VL) [26]. Overall, it is noticeable that *B. subtilis* expression systems are mostly carried out in complex medium but not in defined mineral salt media. However, this is required for establishing high cell density cultures. To enable further improvements in product yield, native auto-regulatory cell density coupling expression systems were utilized in protease-deficient *B. subtilis* strains, such as *B. subtilis* WB600 and WB800, although these strains did not lead to a high overall production yield of recombinant proteins [30].

In addition, laboratory *B. subtilis* strains, such as strain 168 and its derivative 3NA, were used for the bioproduction of valuable bioactive metabolites and enzymes [28,31,32]. Moreover, genome-reduced *B. subtilis* strains allow further potential for optimization due to a clean secondary metabolic background, a lack of extracellular proteases, prophages, and key sporulation genes [33,34]. In this way, genome reduction is a promising strategy to obtain higher protein concentration by partial removal of the production strain chromosome [35]. The most genome-reduced *B. subtilis* strain to date is PG10, which lacks approximately 36% of the genome [36]. In general, genome-reduced strains are mostly used to express difficult-to-produce proteins, as these strains are described to have an increased translation efficiency and lower production of proteases [36]. However, genome reduction also affects the physiological properties of *B. subtilis*, resulting exemplarily in an inability of *B. subtilis* strain PG10 to grow on mineral salt medium, as reported by Lilge and Kuipers [37]. However, Aguilar Suárez et al. [38] showed that the genome-reduced *B. subtilis* strains, such as the *B. subtilis strain* IIG-Bs-27-39, are partly able to grow on minimal medium. Accordingly, production strains are needed that have the advantages of both genome-reduced and wild-type strains, which makes so-called midi-*Bacillus* strains interesting production strains [39].

In this study, the potential of *B. subtilis* with its status as a partially FDA-approved bacterial strain was used for the first time as a host for the bioproduction of the difficult-to-produce bovine α_S1_-casein protein. For this purpose, the potential of controlled genome reduction was combined with the principle of target protein accumulation in inclusion bodies. Therefore, the strains BMV9, a derivative of the non-sporulating *B. subtilis* 3NA, and the genome-reduced midi-*B. subtilis* strain IIG-Bs-20-5-1 were applied. In subsequent high cell density fermentation processes with chemically defined mineral salt medium in 42 L fed-batch bioreactor systems, insights were provided into an upscaling production process for bovine α_S1_-casein as a main protein component of cow’s milk. Finally, zeta potential and particle size were measured for a pre-cleaned *B. subtilis* protein mixture with abundant recombinant casein product and compared to an α_S1_-casein reference protein.

## 2. Materials and Methods

### 2.1. Chemicals, Materials, and Standard Procedures

Chemicals used were purchased from Carl Roth GmbH & Co. KG (Karlsruhe, Germany), Bio-Rad Laboratories, Inc. (Hercules, CA, USA), and Merck KGaA (Darmstadt, Germany). A standard of his_6_-α_S1_ casein was achieved after cultivation of a heterologous *E. coli* production strain BL21 DE3 (Gold), followed by protein purification using immobilized metal affinity chromatography (IMAC) with Ni-NTA columns (HisTrap HP, 5 × 5 mL nickel column; GE Healthcare Life Sciences; Chicago, IL, USA) and an ÄKTA chromatography system (GE Healthcare Life Sciences, Chicago, IL, USA). An anti-His tag antibody (mouse) (Bio-Rad Laboratories, Inc., Hercules, CA, USA) was used for Western blot approaches. A bovine α_S1_-casein reference standard (Carl Roth GmbH & Co. KG; Karlsruhe, Germany) was used for comparative analyses of protein properties.

### 2.2. Strains, Plasmids, and Conditions of Cultivation

The strains and plasmids used in this study are provided in Table 1.

### 2.3. Cloning Procedures

Standard molecular methods were conducted as described before by Sambrook and Russell [43]. Oligonucleotides used for cloning procedures are provided in Table S1. The gene sequence of a codon-optimized α_S1_-casein gene version (Figure S1) synthesized by Eurofins Genomics (Ebersberg, Germany) was cloned into the pHT254 plasmid system, allowing an N-terminal his_8_-tag protein fusion and an IPTG-inducible gene expression using a P*_grac_*_100_ promoter region [25]. Therefore, the codon-optimized α_S1_-casein gene (603 bp) was amplified from the previously synthesized plasmid “pEX-A128-alpha S1 casein” with the oligonucleotides Casein_pHT_I_FOR and Casein_pHT_I_REV using Q5-polymerase (New England Biolabs; Ipswich, MA, USA). The plasmid backbone of pHT254 was amplified using the oligonucleotides Casein_pHT_V_FOR and Casein_pHT_V_REV. Purification of PCR products was carried out with the QIAquick PCR and Gel Cleanup Kit (QIAGEN GmbH, Hilden, Germany) according to the manufacturer’s instructions. PCR products were fused using the Gibson Assembly Master Mix from New England Biolabs (Ipswich, MA, USA), according to the manufacturer’s instructions, except for an extended incubation period of up to 3 h of the final reaction mixture [44]. Ligation mixtures were transformed using competent *E. coli* BL21 Gold (DE3) cells. Recombinant clones were screened by colony PCR using *Taq* 5x Master Mix (New England Biolabs, Ipswich, MA, USA), and the cloned expression plasmids were confirmed by Sanger sequencing at Eurofins GmbH (Ebersberg, Germany).

### 2.4. Bioproduction of Recombinant Bovine α_S1_-Casein

In shake flask cultivations, the pre-culture was prepared in LB medium (10 g/L tryptone, 5 g/L yeast extract, and 5 g/L NaCl) supplemented with 5 µg/mL of chloramphenicol as a selection marker and was cultivated at 37 °C and 120 rpm overnight in an incubator shaker (Newbrunswick^TM^/Innova^®^ 44, Eppendorf AG, Hamburg, Germany). For the main culture, cells from the pre-culture were inoculated in TB medium (24 g/L yeast extract, 20 g/L tryptone, 5 g/L glycerol, 0.017 M KH_2_PO_4_, 0.072 M K_2_HPO_4_) supplemented with chloramphenicol (5 µg/mL) as a selection marker to an optical density (OD_600nm_) of 0.1. The subsequent cultivation process was performed as described before. The target gene expression was induced at an OD_600nm_ of about 1.0 using 1 mM (*w*/*v*) of sterile filtered isopropyl-β-D-thiogalactopyranosid (IPTG). The induction was carried out for 20 h.

Bioreactor cultivations were carried with the genome-reduced *B. subtilis* strain IIG-Bs-20-5-1 according to descriptions from Klausmann et al. [27]. The initial pre-culture was performed in L -medium. The second pre-culture was inoculated in mineral salt medium, consisting of 5.5 g/L glucose × H_2_O, 4 g/L Na_2_HPO_4_, 14.6 g/L KH_2_PO_4_, 4.5 g/L (NH_4_)_2_SO_4_, 0.2 g/L MgSO_4_ × 7 H_2_O, 3 mL/L trace element solution (TES) and 25 g/L (*w*/*v*) glucose. TES contained 40 mM Na_3_citrate, 5 mM CaCl_2_, 50 mM FeSO_4_, and 0.6 mM MnSO_4_ × H_2_O. Media used for fermentation processes were previously described by Willenbacher et al. [45] and Wenzel et al. [46]. Bioreactor cultivations were performed in a 42 L fermenter (ZETA GmbH, Graz/Lieboch, Austria) filled with 12 L batch medium using 25 g/L (*w*/*v*) of glucose as carbon source. The temperature was set to 37 °C, pH to 7.0, and initial stirrer speed to 300 rpm. A minimum dissolved oxygen level of 40% was achieved by modifying the stirrer speed and aeration rate. Initially, the aeration rate during the batch phase was set to 2 L/min and subsequently increased up to 22 L/min after starting the feeding process. Fed-batch phase was started after glucose depletion using a feed solution with 50% (*w*/*w*) glucose. In addition, 1.44 g/L IPTG was added to the feed solution. The bioreactor experiments were performed in biological duplicates.

### 2.5. Sampling and Sample Analysis

Samples were taken at least every two hours after the induction and were analyzed regarding the OD_600nm_ values and the corresponding biomass. The OD_600nm_ was determined using a spectrophotometer (UV-3100 PC; VWR GmbH, Darmstadt, Germany). Cell dry weight was calculated with a correlation factor of 4.31 obtained by drying the biomass for 24 h at 110 °C and weighing the dried cell pellet. The cell pellet was harvested via centrifugation at 4 °C and 10,000× *g* for 10 min (Microcentrifuge 5430R; Eppendorf AG, Hamburg, Germany). A subsequent mechanical cell disruption was carried out using 0.1 mm glass beads (Disruptor Beads 0.1 mm, Scientific Industries, Inc.; New York, NY, USA) or a high-pressure homogenizer (APV 2000; SPX Flow Technology Rosista GmbH, Unna, Germany). After cell disruption, remaining cell components, including the insoluble proteins, were separated from the soluble protein fraction by centrifugation. The resulting pellet was dissolved in the same volume of 8 M urea solution to obtain the insoluble protein fraction. The protein concentration was determined using a Bradford assay (Roti^®^ Quant; Carl Roth GmbH & Co. KG, Karlsruhe, Germany) [47]. The determination of protein sizes was performed using SDS-PAGE with 4–20% polyacrylamide gels (TGX Stain-Free™ FastCast™ Acrylamide Solutions; Bio-Rad Laboratories, Inc., Hercules, CA, USA) and Coomassie staining (Roti^®^-Blue quick; Carl Roth GmbH & Co. KG; Karlsruhe, Germany).

After gel electrophoresis, the gels were used for Western blot analysis using nitrocellulose/filter paper sandwiches (0.45 µm, Bio-Rad Laboratories, Inc., Hercules, CA, USA) and a wet-type blotting system (Bio-Rad Laboratories, Inc., Hercules, CA, USA). The transfer buffer used consisted of 25 mM Tris, 192 mM glycine, and 20% (*v*/*v*) methanol with a pH of 8.3. Gels, membranes, filter paper, and fiber pads were equilibrated and soaked in transfer buffer before assembly. The transfer was performed at 30 V for 1 h. After transfer, the membrane was blocked for 1 h in 10 mL PBS buffer containing 0.1% (*v*/*v*) Tween20 and 3% (*w*/*v*) bovine serum albumin on a shaking plate, before 3 µL of the primary antibody (mouse anti-histidine tag antibody, clone AD1.1.10, Bio-Rad Laboratories, Inc.; Hercules, CA, USA) was added and incubated for 1 h on the shaking plate. The membrane was then rinsed 4 times with deionized water. For detection, 5 mL of the ready-to-use Novex^®^ Chromogenic Substrate (Invitrogen Corporation, Waltham, MA, USA) was added to the membrane and incubated for 3 min. The reaction was stopped by rinsing the membrane with deionized water. The Coomassie-stained gels and Western blot were scanned using a gel documentation system (Quantum ST5; Vilber Lourmat Deutschland GmbH, Eberhardzell, Germany). To detect the amount of produced and his-tagged α_S1_-casein target protein, samples were purified with HisPur™ Ni-NTA Spin Columns (0.2 mL, Thermo Fisher Scientific, Waltham, MA, USA), and the protein amount was subsequently quantified.

### 2.6. Immobilized Metal Affinity Chromatography

For increasing the abundance of the recombinant α_S1_-casein in the *B. subtilis* protein mixture, the bioreactor culture (Section 2.4) was harvested, and the resulting biomass pellets were stored at −20 °C. Subsequently, a thawed cell pellet was resuspended in 10 volume units of 10x ice-cold binding buffer (20 mM Na-phosphate, 500 mM NaCl, 8 M urea, 40 mM imidazole, pH 7.4) supplemented with 1 mM phenylmethylsulfonyl fluoride (PMSF) as a protease inhibitor. Cell disruption was performed in a high-pressure homogenizer (APV 2000, SPX Flow Technology Germany GmbH, Norderstedt, Germany) with five cycles at a pressure of 1400 bar. To separate the insoluble protein fraction, the crude cell extract was centrifuged with a flow rate of 100 mL/min at 4 °C and 25,000× *g* (Heraeus Contifuge Stratos, 3049 (HCT 22.300) rotor, Thermo Fisher Scientific GmbH, Waltham, USA), and the insoluble fraction was resuspended in the same volume of binding buffer. Afterwards, immobilized metal affinity chromatography (IMAC) was performed to increase the abundance of the α_S1_-casein target protein in the *B. subtilis* protein mixture, using an automated chromatography system (ÄKTA™ start; Cytiva Europe GmbH, Freiburg, Germany). A pre-packed 20 mL HisPrep™ FF 16/10 column preloaded with Ni Sepharose™ 6 Fast Flow was used to capture the recombinant protein. The column was equilibrated with five column volumes (CV) of binding buffer. Unbound proteins were washed out with approximately 10 CV of binding buffer. The bound protein fraction, including the α_S1_-casein fraction, was eluted with a one-step gradient (100%) of elution buffer (20 mM Na-phosphate, 500 mM NaCl, 8 M urea, 500 mM imidazole, pH 7.4). The pooled fractions were processed to remove imidazole with buffer exchange using PD-10 desalting columns (Cytiva Europe GmbH, Freiburg, Germany). The recovery procedure was performed in technical duplicates.

### 2.7. Quantification of Intracellular α_S1_-Casein

The protein concentration at different time points and the purity was determined in the insoluble fraction of the intracellular proteome using GelAnalyzer 23.1.1 (available at www.gelanalyzer.com by Istvan Lazar Jr., PhD and Istvan Lazar Sr., PhD, CSc). Samples were taken after induction, and the his-tagged α_S1_-casein was purified using 0.2 mL His-Trap Spin Columns (Figure S4). SDS-PAGEs (Figure S4) were scanned at 600 dpi, and band intensities were quantified. For each lane, the intensity of the target band and the total intensity of all visible bands were measured. Automatic band detection was performed, and thresholds were adjusted to accurately capture all visible bands while excluding background artifacts. The relative intensity of the target band was calculated as follows:

(1) $$\mathrm{Relative}\;\mathrm{Intensity}=\frac{Intensity\;of\;Target\;Band}{Total\;Lane\;Intensity}$$

The amount of the target protein was determined by multiplying the relative intensity by the total protein amount:

Target Protein Amount = Relative Intensity × Total Protein Amount(2)

(3) $$\mathrm{Volumetric}\;\mathrm{Protein}\;\mathrm{Content}=\frac{Target\;Protein\;Amount}{Volume\;(sampled\;cell\;suspension)}$$

As a reference protein, an in-house expressed and purified His(6)-α_S1_-casein standard from Wang et al. [20] was used. All measurements were performed in duplicates.

### 2.8. Data Analysis

For the estimation of production performances, the yield of product per biomass (*Y_P/X_*), product per substrate (*Y_P/S_*), biomass per substrate (*Y_X/S_*), and the intracellular volumetric casein titer were calculated using the equations below. Therefore, the parameters biomass (*X*), glucose as substrate (*S*), and casein as product (*P*) were used.

(4) $$Y_{P/X}=\frac{P}{X}\;|P=P_{max}$$

(5) $$Y_{P/S}=\frac{P}{S}\;|P=P_{max}$$

(6) $$Y_{X/S}=\frac{X}{S}\;|X=X_{max}$$

### 2.9. Zeta Potential and Particle Size Distribution

Zeta potential and particle size were measured with a Zetasizer Nano-ZS (Malvern Instruments Ltd., Malvern, UK) and evaluated with the corresponding Zetasizer Software 7.2. Measurements were carried out in triplicate at 25 °C in disposable folded capillary cells. Measurements were exerted at a laser wavelength of 633 nm. Zeta potential determination was applied with an angle of 90°. Particle size was determined via dual-angle dynamic light scattering (DLS) at 13° and 173°. The measurement is dependent on laser scattering, and accordingly the refractive index of the solution and dispersant must be considered. A value of 1.33 was chosen for water and 1.57 for α_S1_-casein, which is a generic refractive index used for proteins [48]. Protein solutions with a concentration of 0.2% (*w*/*w*) were used for measurements. Changes in pH were performed stepwise from pH 7 to pH 3.

## 3. Results

### 3.1. Bioproduction of α_S1_-Casein in B. subtilis Strains BMV9 and IIG-Bs-20-5-1

**Figure 1:**
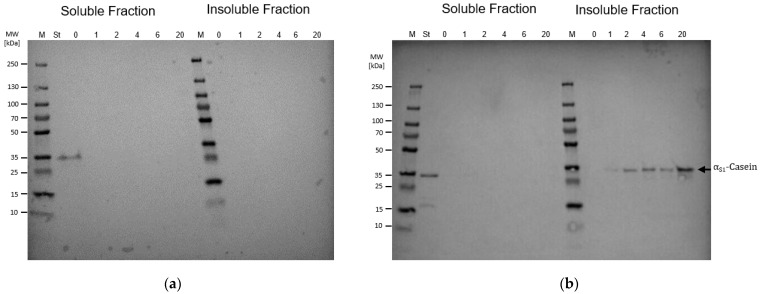
Comparative α_S1_-casein production using different *B. subtilis* strains. Western blot analyses of *B. subtilis* strains BMV9 (**a**) and IIG-Bs-20-5-1 (**b**) carrying the IPTG-inducible pHT254 plasmid system for heterologous α_S1_-casein gene expression. Both the soluble and insoluble intracellular protein fractions were sampled at different time points immediately before (0′) and 1, 2, 4, 6, and 20 h after the induction of target gene expression. (M = Marker; St = α_S1_-casein standard from *E. coli*).

Since, to the best of our knowledge, *B. subtilis* has not yet been established as a host for the heterologous bioproduction of casein proteins, strains were selected for analysis of their α_S1_-casein production efficiency: the non-sporulating and surfactin-producing strain BMV9, established for high cell density fermentation processes, and the surfactin-producing genome-reduced midi-*Bacillus* strain IIG-Bs-20-5-1 [40,41]. In a next step, a codon-optimized α_S1_-casein gene was cloned into the expression plasmid pHT254 controlled by the IPTG-inducible P_grac100_ promoter system [25]. The corresponding strains BMV9 and IIG-Bs-20-5-1 carrying the pHT254-α_S1_-casein plasmid were grown in shake flasks with TB medium. Upon reaching an OD_600nm_ of 1.0–1.2 (0.23–0.27 g_CDW_/L), α_S1_-casein production was induced with 1 mM IPTG, and biomass samples were collected for protein detection. Figure 1 provides an overview using SDS-PAGE and Western Blot analyses for samples taken immediately before and 1, 2, 4, 6 and 20 h after induction of target gene expression. The respective cell growth behavior of both *B. subtilis* production strains, BMV9 and IIG-Bs-20-5-1, carrying the pHT254-α_S1_-casein plasmid, and SDS-PAGEs showing the intracellular protein content during the induction process were provided in Figure S2.

In more detail, both the soluble and insoluble protein fractions were applied in the SDS-PAGE. Interestingly, no induced α_S1_-casein expression pattern could be detected in the soluble protein fraction in either strain, while his-tagged α_S1_-casein (theoretical molecular mass: 27.24 kDa) was only available in the insoluble protein fraction of strain IIG-Bs-20-5-1 using Western blot analyses with a C/N-terminal anti-His-tag antibody. The protein size corresponded to the applied casein standard by Wang et al. [20], which also carried a His-tag and had a similar protein sequence and theoretical molecular weight. The theoretical molecular mass of the His-tagged α_S1_-casein was at about 27.24 kDa, although the target protein was consistently detected at ~35 kDa. In contrast to the accumulation of the his-tagged α_S1_-casein target protein in the insoluble protein fraction of the genome-reduced midi-*B. subtilis* strain IIG-Bs-20-5-1, no α_S1_-casein protein signal could be found in the soluble and insoluble protein fraction of the strain BMV9 using Western blot analyses. The detection of the his-tagged α_S1_-casein reference protein excluded any technical issues.

### 3.2. High Cell Density Bioreactor Cultivation for Upscaled α_S1_-Casein Production

**Figure 2:**
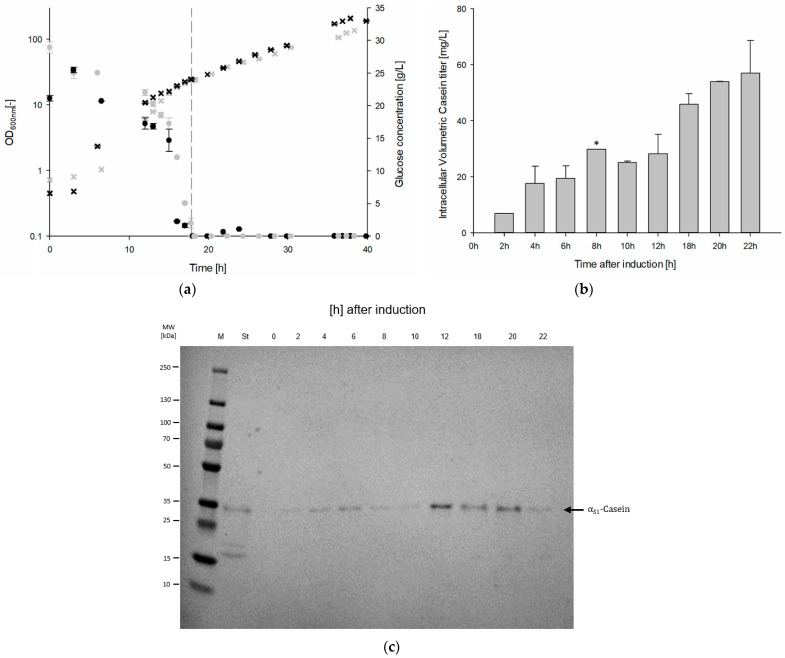
Fed-batch bioreactor cultivation for recombinant α_S1_-casein production. (**a**) The time course of two fed-batch bioreactor fermentations using the α_S1_-casein producing *B. subtilis* strain IIG-Bs-20-5-1 shows the OD_600_ values (black/gray crosses) and glucose concentrations (black/gray circles) in [g/L]. The dotted line indicates the IPTG-mediated induction of α_S1_-casein production. (**b**) The bar chart displays the intracellular volumetric casein titer after induction (* sample 8 h was only in one experiment detectable). (**c**) A representative Western blot using an anti-His tag antibody shows the insoluble protein fraction immediately before (0 h) and 2, 4, 6, 8, 10, 12, 18, 20, and 22 h after induction (St = α_S1_-casein standard).

Since only the genome-reduced *B. subtilis* strain IIG-Bs-20-5-1 allowed for the bioproduction of recombinant α_S1_-casein in shake flask cultivations, this production strain was applied for establishing upscaling of protein production using a fed-batch bioreactor system (Figure 2) [27].

The bioreactor process started with an initial batch cultivation using 25 g/L glucose, followed by a feeding procedure with 50% (*w*/*w*) glucose solution. The precise addition of glucose as the sole carbon source enabled cell growth to be specifically controlled. In this way, a growth rate of µ = 0.1 1/h was planned. To prevent pH fluctuations and nitrogen depletion, the pH was maintained with 19% (*v*/*v*) ammonium solution and 4 M H_3_PO_4_. The time course of the fed-batch bioreactor cultivation is shown in Figure 2a. In the initial batch cultivation, a cell dry weight (CDW) of 5.83 g/L was achieved after approximately 18 h with a maximum specific growth rate (μ_max_) of 0.45 ± 0.07 1/h. After glucose depletion, a subsequent production procedure was started with feeding and induction (1 mM IPTG) at an OD_600_ of 24.5 (5.83 g_CDW_/L).

The feeding procedure lasted a total of 22 h and ended with a final CDW of 39.29 ± 5.95 g/L. The growth rate (μ_set_) during the feeding procedure was aimed to be at 0.1 1/h, while the growth rate effectively achieved was 0.0871 ± 0.006 1/h, leading to a biomass yield (Y_X/S_) of 0.19 g/g. After 40 h of cultivation, cells were harvested for further analysis and target protein pre-cleaning procedure. Overall, a final product yield (Y_P/X_) of 2.16 mg_casein_/g_CDW_ and a final substrate conversion yield (Y_P/S_) of 0.32 mg_casein_/g_glucose_ were determined.

Figure 2c shows SDS-PAGE and Western blot analyses of the insoluble protein fraction for the samples collected at different time points after induction. The SDS-PAGE analysis revealed no significant increase in the signal intensity, which is consistent with observations described for shake flask cultivations in Section 3.1. In contrast, Western blot analysis allowed the detection of α_S1_-casein target protein already 2 h after induction. A subsequent quantification allowed the determination of the intracellular volumetric casein titer during the bioprocess (Figure 2b). Overall, the intracellular volumetric casein content increased over the course of cultivation, with a maximum α_S1_-casein content of 56.9 ± 11.6 mg/L determined 22 h after induction.

### 3.3. Increasing the Abundance of Recombinant Bovine α_S1_-Casein in Insoluble B. subtilis Protein Fraction

**Figure 3:**
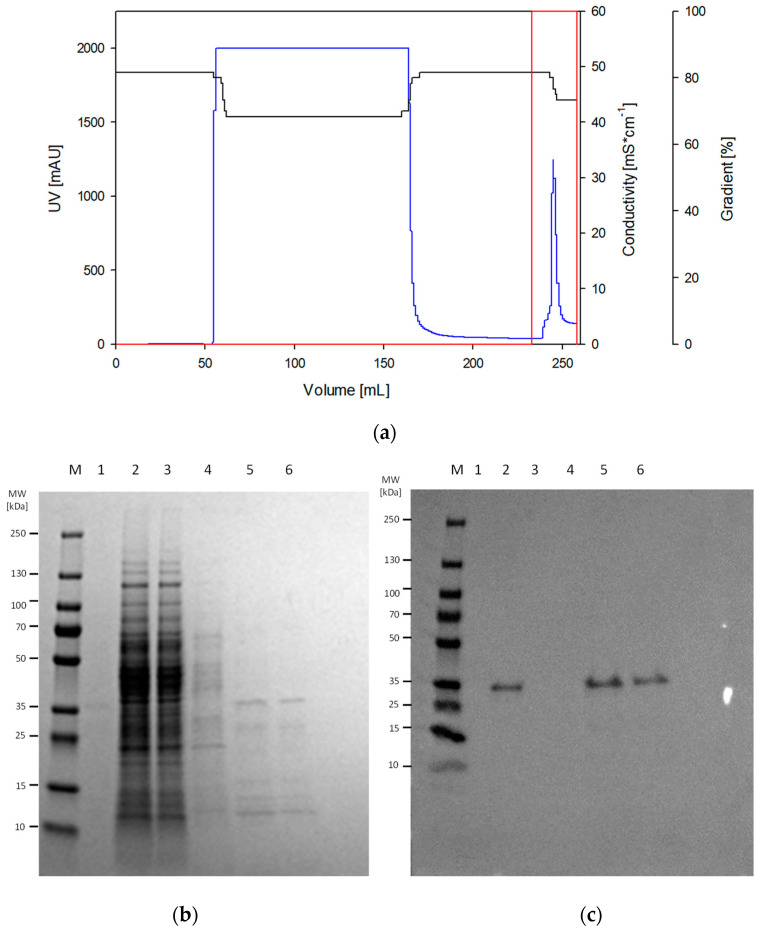
Pre-cleaning of recombinant α_S1_-casein in the insoluble *B. subtilis* protein fraction by IMAC approach. (**a**) The chromatogram displays the affinity chromatography addressing the his(8)-tagged α_S1_-casein from a fed-batch bioreactor cultivation broth. The UV signal (blue line), the conductivity (black line), and the gradient of the elution step (red line) are plotted. (**b**) SDS-PAGE and (**c**) Western blot analysis showing the insoluble protein fractions after cell lysis (lane 2) as well as flow-through, wash, and elution fractions 1 and 2 from the IMAC process (lanes 3–6). Equivalent amounts of total protein (6 μg for lanes 1–4; 4 μg for lanes 5–6) were loaded onto the gel, and protein bands were visualized by Coomassie brilliant blue staining. For the Western blot approach, an anti-His tag antibody was used.

In total, 5 L cultivation broth from the bioreactor cultivation process described in Section 3.2 was taken for cell separation, which resulted in a cell pellet of approximately 30 g for further processing. Since an accumulation of α_S1_-casein was only found in the insoluble protein fraction, this fraction was applied after cell disruption for increasing the abundance of α_S1_-casein using ÄKTA-based immobilized metal affinity chromatography (IMAC) as a pre-cleaning procedure. Figure 3a shows the pronounced peak in the UV signal of the chromatogram (blue line), which occurred after elution with a one-step gradient and proved the successful pre-cleaning of the recombinant α_S1_-casein containing the insoluble protein fraction. A corresponding protein signal of approximately 35 kDa could be observed in subsequent SDS-PAGE and Western blot analysis, which confirmed the presence of the expressed recombinant α_S1_-casein target protein (Figure 3b,c). Since the purification of target proteins from insoluble protein fractions requires a complex downstream processing, the ambition of the study was an increase in the molecular abundance of the α_S1_-casein target protein within the *B. subtilis* protein mixture, which could be confirmed in Figure 3b. Accordingly, a dominant part of the *B. subtilis* proteins could be removed, while some impurities were still present in the SDS-PAGE. Overall, by comparing the signal intensity of the total proteins within an SDS PAGE lane and the casein target protein, a purity estimation of 46.5% was achieved. A final Bradford analysis using the collected fractions after elution could show an overall target protein concentration of about 0.072 mg/mL. Accordingly, a total product per biomass yield (Y_P/X_) of 1.6 mg_Casein_/g_CDW_ could be reached.

### 3.4. Comparative Analysis of Zeta Potential and Particle Size Distribution

Even though the IMAC approach only served as a pre-cleanup approach leading to a higher abundance of the α_S1_-casein target protein, further comparative studies were carried out in terms of important parameters of zeta potential and particle size distribution using native bovine α_S1_-casein as a reference.

**Figure 4:**
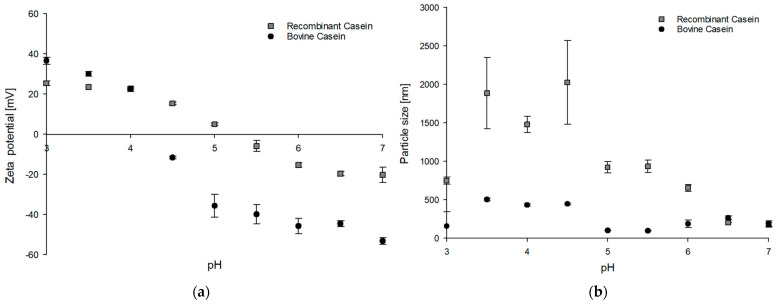
Comparative characterization of the protein properties of the bovine α_S1_-casein reference and pre-cleaned recombinant α_S1_-casein protein mixture. (**a**) Plot of the zeta potential and (**b**) particle size of pre-cleaned recombinant (gray squares) and bovine α_S1_-casein (black circles) across different pH values, ranging from pH 3 to 7. The analyses were performed in triplicates.

For this purpose, the zeta potential and particle size of both the pre-cleaned recombinant α_S1_-casein protein mixture and the bovine α_S1_-casein reference were analyzed in a pH range of 3 to 7. In more detail, the highest zeta potential values of 36.5 mV and 25.3 mV were observed at a pH of 3 for the bovine and recombinant α_S1_-casein protein mixtures, respectively. In addition, the higher the pH values tested, the lower the zeta potential values of both α_S1_-casein sample types with −20.3 mV (pre-cleaned recombinant α_S1_-casein protein mixture) and −53.2 mV (bovine α_S1_-casein reference) at a pH of 7. The isoelectric point of bovine α_S1_-casein reference is between pH 4.0 and 4.5, whereas it is between 5.0 and 5.5 for pre-cleaned recombinant α_S1_-casein protein mixture. An overview of the zeta potentials measured for both types of samples is provided in Figure 4a.

In contrast to the zeta potential, the determination of particle sizes at different pH values varied clearly between both sample types, especially in the pH range between 5.0 and 3.5. At pH 4.5, the *B. subtilis* protein mixture with accumulated recombinant α_S1_-casein displayed a significantly larger particle size of 2025 nm compared to the bovine α_S1_-casein reference of 431.6 nm, indicating a 4.7-fold increase. Overall, bovine casein reference maintained a consistent particle size of 263.62 ± 147.61 nm across the entire pH range, although with increased variability at lower pH values. The *B. subtilis* protein mixed with abundant recombinant α_S1_-casein exhibited its largest particle size of 1369.20 ± 576.41 nm between pH 3.5 and 4.5, followed by a decline as pH increased. Accordingly, the values of both bovine reference and the pre-cleaned recombinant α_S1_-casein protein mixture are comparable at a pH range of 6.5 and 7 (187.75 ± 13.25 nm for pre-cleaned recombinant α_S1_-casein protein mixture and 227.05 ± 37.55 nm for bovine α_S1_-casein reference).

## 4. Discussion

Finding new expression strains is a crucial step in the development of new biotechnology-based principles for food protein production. In this context, *B. subtilis* provides a good baseline as a well-established expression host for recombinant protein production, as summarized by Souza et al. [49], and is partially accepted as a GRAS microorganism. Here, genome minimization was previously described as a promising approach for developing platform strains for difficult-to-produce proteins with a clean secondary metabolic background [33,34]. However, as genome reduction also affects physiological properties of *B. subtilis*, as shown for *B. subtilis* strain PG10, the most genome-reduced *B. subtilis* strain to date [36]. Accordingly, *B. subtilis* strains revealing less genome reduction seem to be promising for future bioproduction processes, making so-called midi-*Bacillus* strains interesting production strains for difficult-to-produce bioproducts [39].

In this study, the potential of the genome-reduced midi-*B. subtilis* strain IIG-Bs-20-5-1 was used for the recombinant bioproduction of the difficult-to-produce bovine α_S1_-casein [41]. The *B. subtilis* strain BMV9, a sporulation-deficient and surfactin-producing derivative of *B. subtilis* strain 3NA, was used as a reference strain. Interestingly, although the selected *B. subtilis* strains differ in about 10% of genome complexity, only midi-*B. subtilis* strain IIG-Bs-20-5-1 allowed a detectable intracellular α_S1_-casein bioproduction, while absolutely no target protein detection was observed for *B. subtilis* strain BMV9 (Figure 1). The partial genome reduction in the *B. subtilis* strain IIG-Bs-20-5-1 includes the deletion of genes encoding for the biosynthesis of the non-ribosomally produced metabolites plipastatin, bacilysin, and polyketides, but also lacks the biosynthesis of the antimicrobial substances subtilosin A, kanosamine, and the *Bacillus* toxin Sdp, but also several genes encoding for prophages and the alternative sigma factors SigE and SigG [41,50]. In contrast to the strain IIG-Bs-20-5-1, the 3NA derivative BMV9, which was optimized for high cell density cultivations, features a point mutation in the *spo0A* gene locus and a 33 bp-comprising elongation of the *abrB* gene. These point mutations make the *B. subtilis* strain BMV9 a promising surfactin production strain in high cell density fermentation processes [40]. However, although only the midi-*Bacillus* strain allowed the bioproduction of α_S1_-casein as the target protein, it is not clear whether the sole reduction in metabolic side-streams or deletion of certain genes achieved the bioproduction. Nevertheless, this work provides *B. subtilis* the first time as a production strain for recombinant casein bioproduction. However, especially genome-reduced *B. subtilis* strains were previously described as an attractive chassis for recombinant protein production. In this context, Schilling et al. [34] demonstrated that genome reduction in *B. subtilis* leads to significantly improved secretion and protein activation of disulfide-linked proteins. Aguilar Suárez et al. [38] also showed that genome reduction promotes the production of recombinant proteins, including extracellular protein IsaA and extracellular staphylococcal target proteins. Furthermore, it was shown that a guided genome reduction as in strain IIG-Bs-27-39 offers significant advantages in terms of growth, metabolic efficiency, and stability of protein production. These results provide the way for the further increase in recombinant casein production [38].

In the subsequent upscaling of bioproduction processes, the cultivation of a genome-reduced *B. subtilis* strain using large-scale fed-batch bioreactor fermentation was described for the first time in the scientific literature (Figure 2), showing the potential of genome-reduced *Bacillus* strains as viable platform organisms for large-scale, difficult-to-produce protein biosynthesis. This scalability is crucial for the transition from laboratory-scale experiments to industrial applications, highlighting the broad industrial relevance of *B. subtilis* in biotechnological applications.

However, since an α_S1_-casein accumulation in the insoluble protein fraction was found, a subsequent complete purification needs a laborious downstream purification. Nevertheless, a first pre-cleanup was performed using urea as a chaotropic agent for denaturing proteins in inclusion bodies and ÄKTA-mediated affinity chromatography for targeting the his-tagged α_S1_-casein. Based on this pre-cleanup, an accumulation of the α_S1_-casein with a purity of 46.5% and a titer of 2.16 mg_Casein_/g_CDW_ could be achieved (Figure 3). Further improvements of the titer might be available in protease-deficient *B. subtilis* strains. In this context, Duanis-Assaf et al. [51] stated the capability of *B. subtilis* to degrade other casein subunits (κ-casein). Since the α_S1_-casein target protein was only detectable in the insoluble protein fraction, *B. subtilis* proteases, like Clp proteases, might actively degrade the unstructured α_S1_-casein version [52]. In another study, Airaksinen et al. [53] described the intracellular bioproduction of *Chlamydia pneumoniae* proteins in secretory protease-deficient *Bacillus subtilis* strains WB600 and IA289 with localization of the proteins MOMP and Omp2 in the inclusion bodies and Hsp60 in the soluble cytosolic fraction [53]. However, the intracellular accumulation of α_S1_-casein also provides the basis for the formation of micelle-like structures if co-expression of further casein subunits occurs.

In comparison, the results in our work show the successful production of recombinant bovine α_S1_-casein in *B. subtilis*, a species that is generally recognized as safe (GRAS) [54]. This makes the production of food proteins in *B. subtilis* particularly attractive. In combination with a robust cell growth, the use of high cell-density fermentation processes and further flexible genetic engineering approaches, *B. subtilis* is an ideal candidate for the bioproduction of food proteins. Only the downstream processing with respect to the localization of casein in inclusion bodies appears to require further research in order to be able to make final recipe formulations. Nevertheless, a pre-cleaning by using urea as a chaotropic agent and an immobilized metal affinity chromatography (IMAC) was performed, which led to a target protein purity of 46.5% and a production yield Y_P/X_ of 1.6 mg casein per gram cell dry weight (CDW) (Figure 3a). To further improve downstream processing, modification of parameters such as pH and salt concentration, the addition of dithiothreitol to the elution buffer, or changing the affinity tag might solve this problem [55]. However, the establishment of an individual target protein-specific downstream processing was not the goal of this work and needs to be addressed in future studies.

Nevertheless, to characterize the pre-cleaned recombinant casein protein mixture, zeta potential measurements and particle size determinations were performed using native bovine α_S1_-casein as the reference. In this way, the pre-cleaned recombinant α_S1_-casein protein mixture and native bovine α_S1_-casein revealed significant modulation of surface charge properties with pH. Higher zeta potential values at lower pH were attributed to protonation of acidic residues, with a marked decline as the pH approached the isoelectric point (IEP). The IEP was identified at pH 5.0–5.5 for pre-cleaned recombinant α_S1_-casein protein mixture and at pH 4.0–4.6 for native bovine α_S1_-casein reference, consistent with previous studies [56,57]. Beyond the IEP, the stabilization of surface charge at higher pH may reflect maximum charge repulsion or new intermolecular interactions such as hydrogen bonding or van der Waals forces [58]. Notably, native bovine α_S1_-casein exhibited more negative zeta potential values at higher pH compared to the pre-cleaned recombinant protein version.

In contrast, the pre-cleaned protein mixture with abundant recombinant bovine α_S1_-casein displayed a sharp increase in particle size between pH 3 and 5, likely due to reduced electrostatic repulsion and increased hydrophobic interactions as net charge decreased, consistent with observations in milk-based systems by Rojas-Candelas et al. [59]. In contrast, the native α_S1_-casein reference protein showed relatively stable particle sizes across pH levels [15].

These results indicate that the remaining *B. subtilis* proteins in the pre-cleaned recombinant casein mixture significantly affect the aggregation behavior of the produced α_S1_-casein, leading to a greater tendency to aggregate compared to their native bovine reference substance. Furthermore, the increased particle size variability observed in the pre-cleaned recombinant casein protein mixture at lower pH values could be due to partial aggregation or denaturation of *B. subtilis* proteins combined with the casein target protein, which exposes hydrophobic cores and leads to unpredictable aggregation patterns [60,61]. Accordingly, more efficient downstream processing for casein protein products needs to be established, as the observations in Figure 4 indicate that the casein protein properties and aggregation behavior in response to pH changes are significantly different between the pre-purified recombinant casein protein mixture and the reference casein substance.

## 5. Conclusions

In this study, the potential of using *B. subtilis* as a host for the recombinant production of bovine α_S1_-casein was successfully demonstrated, providing important insights into sustainable food production systems. The research highlights the possibility of genome-reduced *Bacillus* strains in the production of recombinant proteins, with particular focus on α_S1_-casein, an important milk protein. Key findings include the successful expression and initial pre-cleaning of α_S1_-casein with a yield of 1.6 mg_casein_/g_CDW_. The study also highlights challenges associated with the stability of the protein and emphasizes the need for further optimization to prevent possible degradation. For the first time, a fed-batch bioreactor approach of a genome-reduced *B. subtilis* strain for α_S1_-casein bioproduction was carried out. The influence of *B. subtilis* proteins in the pre-cleaned recombinant α_S1_-casein protein mixture was shown in a comparative analysis of zeta potential and particle size between using native bovine α_S1_-casein as a reference. In this way, the pre-cleaned recombinant α_S1_-casein protein mixture was more prone to aggregation at lower pH values than the native α_S1_-casein reference protein. Overall, this research supports the viability of using genome-reduced *B. subtilis* for the production of α_S1_-casein, contributing to more sustainable and resilient food production systems. The results are consistent with the broader goals of developing microbial expression systems for the bioproduction of nutritious and sustainable food, which are in line with current efforts to address global food security challenges.

## Figures and Tables

**Table 1:** Bacterial strains and plasmids used in this study.

Strains and Plasmids	Genotype	Reference
*B. subtilis*		
BMV9	*spo0A3*; Δ*manPA*; *sfp*^+^	Strain collection; [40]
IIG-Bs-20-5-1	P*_mtlA_*-*comKS; trp*^+^; *sfp*^+^; Δ[SPβ]; Δ[*skin*]; Δ[PBSX]; Δ[*proΦ1*]; Δ[*proΦ2*]; Δ[*proΦ3*]; Δ[*proΦ4*]; Δ[*proΦ5*]; Δ[*proΦ6*]; Δ[*proΦ7*]; Δ[*pks*]; Δ[*manPA-yjdF-yjdGH*I*-yjzHJ*]; Δ[*sboAX-albABCDEFG*]; Δ*ppsABCDE*; Δ*bacABCDEF*; Δ[*ytpAB-ytoA*]; Δ[*sdpABCIR*]; Δ[*bpr-spoIIGA-sigEG*]; Δ[*ntdABC-glcP*]	Strain collection; [41]
*E. coli*		
BL21 DE3 (Gold)	$F^{-};\;\mathrm{dcm};\;\mathrm{ompT}\;;\mathrm{hsdS}(r_{B}^{-}m_{B}^{-}$) *gal*; λ(DE3)	[42] New England Biolabs (Ipswich, MA, USA)
Plasmids		
pEX-A128-alpha S1 casein	pUC ori, *amp^R^*	Eurofins Genomics (Ebersberg, Germany)
pHT254	*cm^R^; B. subtilis*/*E. coli* shuttle vector for regulated gene expression (P_grac100_; *lacI*; *repA*, *amp^R^*, ColE1*, N-terminal his_8_-tag)	(MoBiTec GmbH, Goettingen, Germany)
pET22b-bc	(*bla*; *lacI*; P_T7_) pET22b(+) with *Nde*I/*Hin*dIII fragment containing bovine α_S1_-casein encoding gene	[20]
pHT254-αS1_Casein	*cm^R^;* pHT254 vector containing bovine α_S1_-casein encoding gene	This study

## Data Availability

The raw data supporting the conclusions of this article will be made available by the authors on request.

## Supplementary Materials

The following supporting information can be downloaded at: https://www.mdpi.com/article/10.3390/microorganisms13010060/s1, Table S1: List of oligonucleotides used for this study; Figure S1: Gene sequence of the *B. subtilis* codon optimized α_S1_-casein; Figure S2: Time-course of shake flask cultivation of *B. subtilis* strains BMV9 (black circle) and IIG-Bs-20-5-1 (gray triangles) containing expression plasmid pHT254-α_S1_-casein in TB-medium until t = 21 h of cultivation after IPTG-mediated induction of target gene expression; Figure S3: SDS-PAGEs presenting the soluble and insoluble protein fractions after inducing the casein gene expression in the *B. subtilis* strains BMV9 (a) and IIG-Bs-20-5-1 (b); Figure S4: SDS-PAGE analyses of IIG-Bs-20-5-1 carrying the IPTG-inducible pHT254 plasmid system for heterologous α_S1_-casein gene expression in a fed-batch bioreactor cultivation shows the insoluble protein fraction immediately before (0 h) and 2, 4, 6, 8, 10, 12, 18, 20, and 22 h after induction (M = Marker St = α_S1_-casein standard from *E.coli*); Figure S5: Quantitative SDS-PAGEs of the Fed-batch bioreactor cultivations for recombinant α_S1_-casein production. The SDS-PAGEs (4–20%) show the purified insoluble protein fraction immediately before (0 h) and 2, 4, 6, 8, 10, 12, 18, 20, and 22 h after induction (St = α_S1_-casein standard).
