# Supplementary material for: Recombinant Production of Bovine αS1-Casein in Genome-Reduced Bacillus subtilis Strain IIG-Bs-20-5-1
Source: Microorganisms. 2025 Jan 2;13(1):60. doi: 10.3390/microorganisms13010060 (PMC11767299; doi:10.3390/microorganisms13010060)
Supplement: Supplementary file 1 [file microorganisms-13-00060-s001.zip › microorganisms-3398690-supplementary.pdf]

Supplementary Information

Recombinant Production of Bovine α_{S1}-Casein in Genome-Reduced *Bacillus subtilis* Strain IIG-Bs-20-5-1

Lennart Biermann [1], Lea Rahel Tadele [1], Elvio Henrique Benatto Perino [1], Reed Nicholson [2], Lars Lilge [1,*] and Rudolf Hausmann [1]

[1] Institute of Food Science and Biotechnology, Department of Bioprocess Engineering, University of Hohenheim, Fruwirthstraße 12, 70599 Stuttgart, Germany; lennart.biermann@uni-hohenheim.de (L.B.); lea.tadele@uni-hohenheim.de (L.R.T.); eperino@uni-hohenheim.de (E.H.B.P.); rudolf.hausmann@uni-hohenheim.de (R.H.)

[2] Motif FoodWorks, Inc., 27 Drydock Ave, Boston, MA 02210, USA; reed.a.nicholson@gmail.com

* Correspondence: lars.lilge@uni-hohenheim.de; Tel.: +49-711-459-24736

Table S1: List of oligonucleotides used for this study.

Name	Sequence (5’ → 3’)	Purpose
Casein_pHT_V_FOR	GCTGTGGCATCACCATCACCATCACCATCACT	Vector Amplification for Gibson Assembly
Casein_pHT_V_REV	GCTTAGGTCTCATTGATCCTTCCTCCTTTATATGGGAATTG	Vector Amplification for Gibson Assembly
Casein_pHT_I_FOR	GGATCAATGAGACCTAAGCATCCGATTAAACATCAAG	Insert Amplification for Gibson Assembly
Casein_pHT_I_REV	TGGTGATGCCACAGCGGCATCGTTGTC	Insert Amplification for Gibson Assembly

5´-ATGAGACCTAAGCATCCGATTAAACATCAAGGCCTGCCTCAAGAAGTTCTGAA
TGAAAATCTGCTGAGATTCTTTGTGGCACCGTTTCCTGAGGTGTTCGGCAAAGA
AAAAGTTAATGAACTGTCAAAAGATATTGGCTCAGAATCAACAGAAGATCAAGCA
ATGGAAGATATTAAACAAATGGAAGCTGAAAGCATTAGCTCATCAGAAGAGATTG
TTCCGAATAGCGTGGAACAAAAACATATTCAAAAGGAGGATGTTCCGAGCGAAA
GATATCTGGGCTATCTTGAACAACTTCTGAGACTTAAAAAGTATAAGGTTCCGCA
ACTTGAAATTGTTCCGAATAGCGCAGAAGAGAGACTTCATAGCATGAAAGAAGG
CATTCATGCGCAACAGAAAGAACCGATGATTGGAGTTAATCAAGAACTTGCGTA
TTTTTATCCGGAACTGTTTAGACAATTCTATCAACTGGATGCGTATCCGTCAGGC
GCTTGGTATTACGTTCCGCTGGGAACACAATATACAGATGCTCCGTCTTTTAGC
GATATTCCGAATCCGATTGGCTCTGAAAACTCTGAAAAGACAACGATGCCGCTG
TGGTAA-*3´*

Figure S1. Gene sequence of the *B. subtilis* codon optimized α_{S1}-casein.

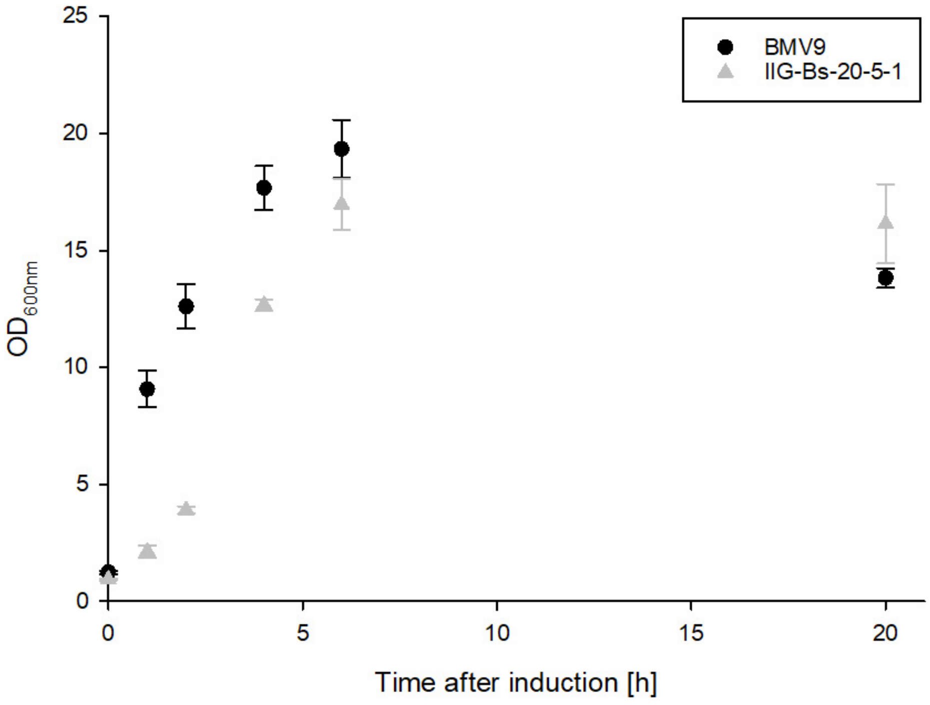

Figure S2. Time-course of shake flask cultivation of *B. subtilis* strains BMV9 (black circle) and IIG-Bs-20-5-1 (grey triangles) containing expression plasmid pHT254-α_{S1}-casein in TB-medium until t = 21 h of cultivation after IPTG-mediated induction of target gene expression.

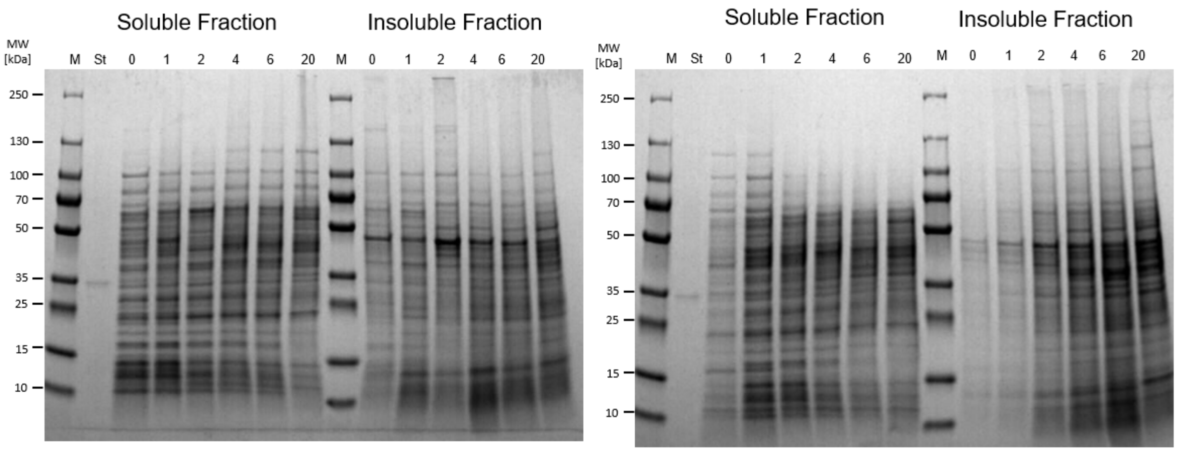

(a) (b)

Figure S3. Comparative α_{S1}-casein production using different *B. subtilis* strains. SDS-PAGE analyses of *B. subtilis* strains BMV9 (a) and IIG-Bs-20-5-1 (b) carrying the IPTG-inducible pHT254 plasmid system for heterologous α_{S1}-casein gene expression. Both the soluble and insoluble protein fractions were sampled at different time points immediately before (0´) and 1, 2, 4, 6 and 20 h after induction of target gene expression. (M = Marker; St. = α_{S1}-casein standard from *E. coli*).

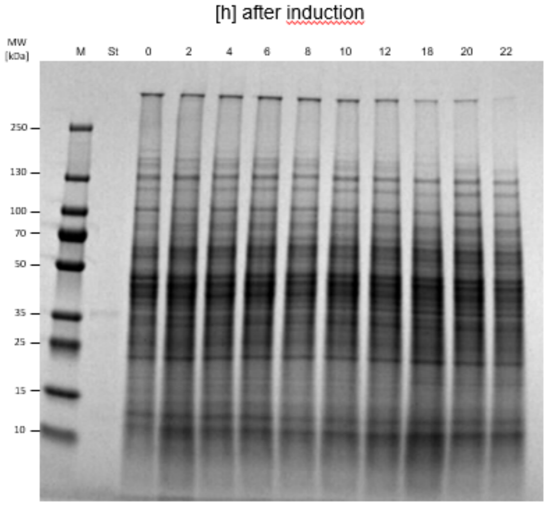

Figure S4. SDS-PAGE analyses of IIG-Bs-20-5-1 carrying the IPTG-inducible pHT254 plasmid system for heterologous α_{S1}-casein gene expression in a fed-batch bioreactor cultivation shows the insoluble protein fraction immediately before (0 h) and 2, 4, 6, 8, 10, 12, 18, 20, 22 h after induction (M = Marker St = αS1-casein standard from *E.coli*).

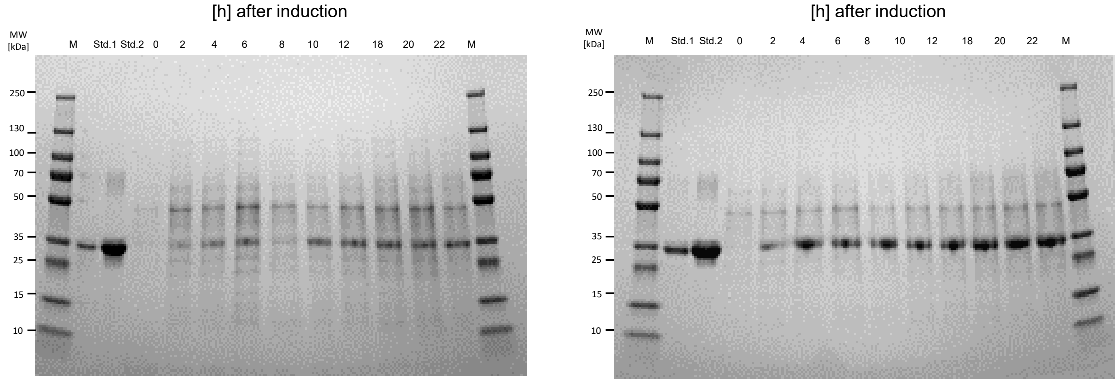

Figure S5. Quantitative SDS-PAGEs of the Fed-batch bioreactor cultivations for recombinant α_{S1}-casein production. The SDS-PAGEs (4-20%) show the purified insoluble protein fraction immediately before (0 h) and 2, 4, 6, 8, 10, 12, 18, 20, 22 h after induction (St = α_{S1}-casein standard).
